# Notes from Chautauqua County

**Published:** 1895-09

**Authors:** 


### NOTES FROM CHAUTAUQUA COUNTY.

The physicians of Chautauqua county are organising against dishonest debtors, as will be observed by the following circular:

Sherman, N. Y., August 1, 1895.

Dear Doctor,—Knowing by experience the amount of charitable work all physicians do (and willingly), several of us have agreed to help one another to organise a plan to avoid serving "dead beats." The plan is this: every four months each member is to send to the undersigned the names of all parties that have played the "dead beat act" or refused to pay proper accounts. The names from all over the county are to be arranged in alphabetical order and printed on good paper in a way to be of easy reference. A new list will be prepared January 1st, May 1st and September 1st of each year, if this receives the support it should have. Who among us would not many a time rather have given a dollar and been able to sleep, than ride in some storm to find that the newcomer never had paid a doctor and probably never would ? There is nothing in this to prevent anyone attending any person they desire ; but all who wish can know whether their call is to parties who are not in the habit of paying. If this meets with your approval, sign "yes" on the enclosed card, with the other blanks filled, and mail at once, then make out your list and send it with 50 cents to me as soon as possible, and by September 1st I will try to have all lists ready.

Fraternally, C. A. ELLIS,
*Sec'y Chautauqua Co. Medical Society.*

---

CALLING THEMSELVES DOCTORS.—The new law fixing the penalty at from $250 to $500, or imprisonment, or both, for any persons practising medicine illegally, or calling themselves "doctor" will probably be enforced in Chautauqua county. At the annual meeting of the Medical Society of the County of Chautauqua, held July 11th, the following motion was unanimously passed :

That the board of censors be authorised to draw upon the treasury for any amount required to investigate the illegal practice of medicine, and to secure the enforcement of the law.

The board of censors is determined to make the most of this opportunity and will proceed to call to account any persons whom they believe are liable to the penalties of the statute.

---

DR. EDWARD AMES, of Kalamazoo, Mich., formerly of Sherman, N. Y., has been spending a part of his vacation in Chautauqua county. He will return home and visit Boston with the Knights Templar.

---

DR. NELSON G. RICHMOND, of Fredonia, has returned from New York, where he has been pursuing a post-graduate course with particular reference to intubation and diseases of the rectum.

---

DR. MORRIS N. BEMUS, of Jamestown, has been appointed pension examiner, in place of Dr. H. P. Hall, deceased.

---

DR. W. A. PUTNAM, of Westfield, is getting to be one of the crack shots of the Westfield Gun Club.

